# The Influence of Oblique Angle Forced Exercise in Surgically Destabilized Stifle Joints Is Synergistic with Bone, but Antagonistic with Cartilage in an Ovine Model of Osteoarthritis

**DOI:** 10.1155/2017/7481619

**Published:** 2017-02-27

**Authors:** Rachel J. Hill, Holly M. Mason, Gavin Yeip, Samer S. Merchant, Aaron L. Olsen, Rusty D. Stott, Arnaud J. Van Wettere, Eadric Bressel, Jeffrey B. Mason

**Affiliations:** ^1^Department of Animal, Dairy and Veterinary Sciences, Center for Integrated BioSystems, School of Veterinary Medicine, Utah State University, Logan, UT, USA; ^2^Department of Animal, Dairy and Veterinary Sciences, Clinical Veterinary Services, School of Veterinary Medicine, Utah State University, Logan, UT, USA; ^3^Department of Bioengineering, College of Engineering, University of Utah, Salt Lake City, UT, USA; ^4^Utah Veterinary Diagnostic Laboratory, School of Veterinary Medicine, Utah State University, Logan, UT, USA; ^5^Department of Kinesiology & Health Science, Utah State University, Logan, UT, USA

## Abstract

Large animal models of osteoarthritis are a necessary testing ground for FDA approval of human medicine applications. Sheep models have advantages over other available large animals, but development and progression of osteoarthritis in sheep is exceedingly slow, which handicaps progress in development of potential treatments. We combined oblique angle forced exercise to increase stress on the stifle, with surgical destabilization to hasten the development of osteoarthritis in ewes. Methods for early detection of clinical signs included radiography, urine, and serum biomarker assays and gait analysis and ex vivo we used microcomputed tomography and macroscopic joint analysis. Our model was able to produce clinically detectable signs of osteoarthritis in a relatively short period (14 weeks). Changes in bone were highly correlated between microcomputed tomography and radiographic analysis and changes in cartilage correlated well between urinary glycosaminoglycan levels and serum aggrecanase analyses. Exercise improved the negative effects of destabilization in bone but exacerbated the negative effects of destabilization in cartilage. These observations suggest that we may need to consider treatments for bone and cartilage separately. These results represent an improved large animal model of osteoarthritis with rapid onset of disease and superior detection of bone and soft tissue changes.

## 1. Introduction

There is a great need for animal models of osteoarthritis (OA). The Food and Drug Administration (FDA) guidelines for preclinical and clinical evaluation of orthopedic agents recommend evaluation in two different species, including one nonrodent, large animal model. Large animal models are necessary for gaining regulatory approval for clinical use in humans. Commonly recommended large animal models of OA include the dog, sheep, goat, or horse.

The cranial cruciate ligament desmotomy (CCLD, commonly referred to as an anterior cruciate ligament transection-ACLT) is one of the earliest and most well-known OA models and is the most commonly used surgical model in OA research [1]. The rationale for using this model is that CCL injury causes joint destabilization, which subsequently leads to posttraumatic OA [2]. This model imitates the degradation of articular cartilage after CCL rupture and has been used extensively in several animal models. Anatomically, sheep and goats are the best animal groups for this model and the stifle in these animals is large enough for easy replication of the technique.

Compared to the more severe meniscectomy model, the OA lesions in CCLD models better replicate the natural disease process, but clinical signs develop more slowly. In addition, in sheep and goats, development and progression of OA is exceedingly slow, which handicaps progress in development and testing of potential treatments for OA. Most sheep joint destabilization experiments report induction of OA between 6 months and one year postoperatively [3, 4]. Those experiments that reported induction of OA sooner (20 weeks) described very mild or no OA changes [5, 6]. Goats have very dense bone and thick cartilage in the stifle joints making analysis of arthritic changes in goats difficult. Skeletally mature sheep have advantages over other available large animal models. Because of their anatomic similarities to humans, sheep have become popular as models for orthopedic research. Sheep have been used to study many musculoskeletal conditions including the treatment of osteoarthritis and osteoporosis, repair of fractures, and limb lengthening [7]. Using a sheep model also enables the use of individuals that are almost genetically identical and many sheep models provide data that has good predictive value for appraisal of arthritis treatments [8].

Forced exercise can accelerate the progression of disease in many OA models and exercise has been demonstrated to exacerbate osteoarthritic changes in sheep [4, 9]. Prolonged, repetitive, and heavy loading of joints results in deformation of articular cartilage and chondrocytes and promotes the initiation and progression of OA [10]. Differences in the magnitude or frequency of cartilage loading can have different effects on chondrocyte activity and can either up- or downregulate synthesis of many macromolecules in the cartilage matrix, many of which are involved in matrix breakdown [11].

Being able to detect OA changes early in the progression of the disease would provide the best opportunity to discover interventions that may be able to modify disease progression prior to the appearance of incapacitating damage to the patient's tissues. The most widely used method for detection of OA in humans is the comparison of joint radiographic images over time. However, radiographs do not efficiently detect OA changes during early progression of the disease. When available, imaging joints with computed tomography (CT) technology can improve detection of early degenerative joint changes [12]. When analyzing joints ex vivo, micro-CT is able to detect even smaller changes than in vivo CT imaging. Several imaging modalities are also available for detection of soft tissue abnormalities/lesions, but like radiographs, these imaging techniques have their own limitations and are subject to human biases during scoring. Many in vitro biomarker assays for soft tissue injury analysis avoid this bias by providing quantitative measures of tissue damage, similar to CT analysis.

In the current pilot experiments, we combined a unique application of forced exercise, with a common surgical destabilization method to hasten the development of OA using CCLD, as this best mimics natural CCL injury. We also used several subjective and quantitative approaches to facilitate detection of OA at its earliest stages.

## 2. Materials and Methods

### 2.1. Sheep

Mature Suffolk-cross ewes, three to four years of age with body condition scores between 3 and 4 and no history of musculoskeletal disease, were used in the current experiments. The sheep were fed hay twice daily and water ad libitum throughout the duration of these studies. The experiments were conducted from mid-April to mid-August, when ewes in Northern Utah are seasonally anestrous. At the time of collection, ewes were determined to be anestrous/acyclic due to the visual lack of active follicular development on the ovaries. All sheep were group housed in large (16 ft × 32 ft) indoor pens and were free to exercise. Housing, surgical procedures, treadmill exercise, gait analysis, radiographic imaging, in vivo sample collection, and monitoring were conducted at the USDA ARS Poisonous Plant Research Laboratory. Pathological analysis was conducted at the Utah Veterinary Diagnostic Laboratory. Micro-CT scanning was conducted at the University of Utah Small Animal Imaging Core facility.

### 2.2. Ethical Treatment of Animals

Animal care and use protocols were developed under the Animal Welfare Act (Nov. 6, 2013, as found in the United States Code, Title 7-Agriculture Chapter 54-Transportation, Sale, and Handling of Certain Animals, sections 2131–2159) and Animal Welfare Regulations guidelines and approved by the Utah State University Institutional Animal Care and Use Committee and were carried out in accordance with all applicable institutional, local, and national guidelines.

### 2.3. Surgical Procedure

Surgical destabilization in the ewes was accomplished using a CCLD. All surgical procedures were conducted at the USDA ARS Poisonous Plant Research Laboratory under approval of the USU IACUC. Briefly, sheep were anesthetized using intravenous ketamine (3 mg/kg IV, MWI Animal Health, Boise, ID) and diazepam (0.5–1 mg/kg IV, MWI Animal Health, Boise, ID) followed by intubation and maintained using 1-2% isoflurane in oxygen. Using standard sterile surgical techniques, an arthrotomy dorsomedial to the medial collateral ligament was performed and the anterior tibial attachment of the medial meniscus and anterior cruciate ligament was identified; forceps were passed behind the CCL to isolate this structure, which was transected with a scalpel blade. Effectiveness of the desmotomy was confirmed based on increased anterior drawer sign. Hemostasis was ensured using electrocautery where necessary. Prior to closure, the joint was lavaged with sterile saline. The joint capsule and associated connective tissue was closed using a simple interrupted patter using 1 Vicryl (Ethicon, Cincinnati, OH). The subcutaneous tissue was closed using 2-0 Vicryl (Ethicon, Cincinnati, OH) on a continuous pattern and the final layer was closed using 3-0 Vicryl (Ethicon, Cincinnati, OH) on the subcuticular tissues to produce a buried suture closure of the skin, also using a simple continuous pattern. Aluminum bandage spray was applied to the wounds (AluSpray, Neogen Animal Safety, Lexington, KY). All animals received cephalosporin antibiotics (West-Ward Pharmaceuticals Corp, West Eatontown, NJ) preoperatively and analgesics (fentanyl patch, 100 ug/hr [Mylan, Canonsburg, PA] and meloxicam, 1 mg/kg IV [MWI Animal Health, Boise, ID]) for 24 hr postoperatively. Following surgical recovery, animals were singly housed in small (8 ft × 10 ft) indoor pens. Three days postoperatively, the animals were moved to group housing in large (16 ft × 32 ft) indoor pens and were free to exercise. Blood samples (10 ml) were collected into plain Vacutainers® (Becton-Dickinson, Franklin Lakes, NJ, USA) tubes without anticoagulants (red top tubes) via jugular venipuncture. Urine samples (10 ml) were collected into plain Vacutainers® tubes via free catch from all animals preoperatively and weekly thereafter.

### 2.4. Exercise

**Figure 1:**
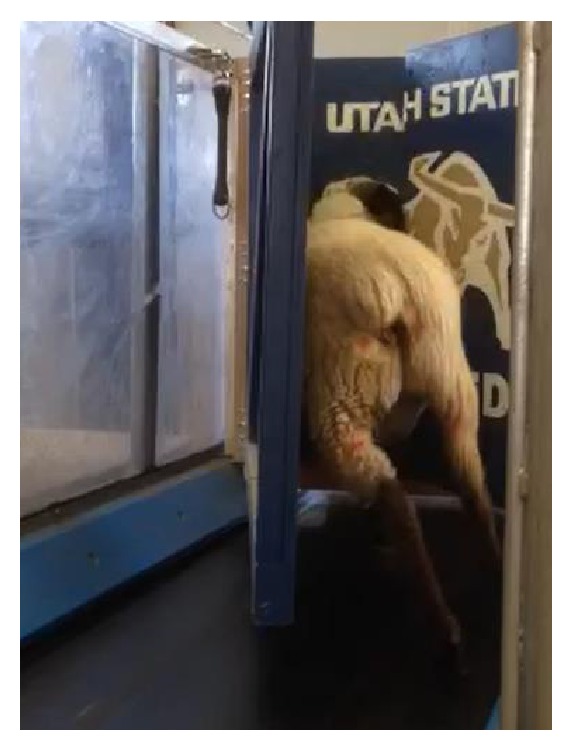
*Exercise*. Moveable treadmill gates forced sheep to move forward at an angle oblique to the movement of the treadmill belt.

Sheep with destabilized and nondestabilized stifle joints were trained to run on the treadmill for two weeks prior to surgery and initiation of experiments. After training, sheep were subjected to forced exercise on a treadmill at 80 M/min for 32 min, six days a week prior to morning feeding. The novel method of forced exercise involved forcing sheep to move at an oblique angle to the treadmill belt. Once the speed of the sheep on the treadmill reached 80 M/min, custom-built treadmill gates were moved into position so that the sheep were forced to run at an oblique angle (35°) to increase mechanical stress on the stifle joint (Figure 1). In sheep with destabilized joints, treadmill running was initiated two weeks postoperatively.

### 2.5. Experimental Design

**Figure 2:**
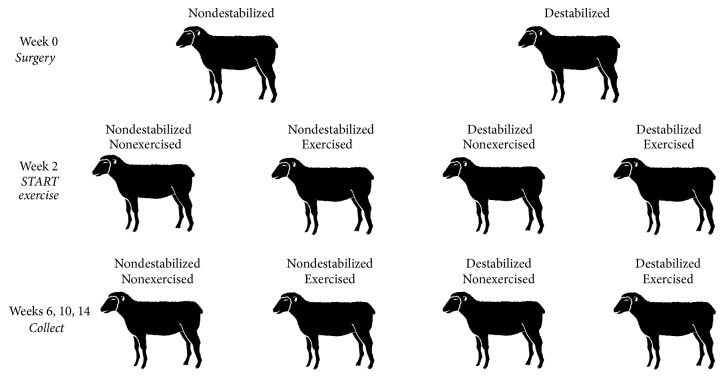
*Experimental Design*. Destabilization surgery (CCLD) took place on Week 0. Treadmill exercise was initiated two weeks postoperatively. Sheep were collected from each experimental group at Weeks 6, 10, and 14.

Twelve ewes were randomly assigned to one of four different treatment groups (three ewes per group) including (1) nonsurgical, unexercised controls, (2) nonsurgical, exercised ewes, (3) surgically destabilized, unexercised ewes, and (4) surgically destabilized and exercised ewes (Figure 2). One animal from each group was euthanized with an overdose of pentobarbital/phenytoin (100 mg/kg, MWI Animal Health, Boise, ID) at six, 10, and 14 weeks after the start of exercise (week two).

### 2.6. Bone Tissue Analysis

#### 2.6.1. Micro-CT

Micro-CT-based morphometric traits in the proximal tibia and the distal femur were measured separately for the four epiphyseal quadrants ex vivo; medial femoral condyle, lateral femoral condyle, medial tibial plateau, and lateral tibial plateau. Micro-CT analysis of the selected treatment groups was performed on an Inveon Trimodality PET/SPECT/CT scanner (Siemens Preclinical Solutions, Knoxville, TN). Exposure time was 1.5 sec with detector settings at 80 kVp and 250 *µ*A. Data was reconstructed with a down sampling factor of 2 onto a 1024 × 1024 × 1454 image matrix using the COBRA software package (Exxim Computing Corporation, Pleasanton, CA). Consequently, the resolution of the reconstructed images was 94.18 *µ*m isotropic. Reconstructed images were analyzed and visualized using Inveon Research Workplace (4.0). A set of three hydroxyapatite (HA) phantoms (750 mg/ccm, 250 mg/ccm, and 50 mg/ccm) were scanned and used for calibration and to compute mean density (MD).

The epiphyseal trabecular total volume (TV, mm^3^), trabecular bone volume (BV, mm^3^), trabecular bone volume fraction (BV/TV, %), specific bone surface (BS/BV, 1/mm), mean trabecular thickness (TbTh, *μ*m), trabecular number (TbN, 1/mm), trabecular separation (TbSp, mm), and trabecular bone pattern factor (TbPf, 1/mm) were determined. Trabecular bone pattern factor, an index of connectivity, where higher values represent a more fragmented lattice, is significantly increased in postreproductive women and is normally increased with age in female mice [13]. Tissue mineral density (TMD) was also determined. Tissue mineral density differs from BMD in that TMD is calculated from the average attenuation value of the bone tissue only and does not include attenuation values from nonbone voxels, as is done for BMD (whether volumetric or areal). Tissue mineral density measurements included MD of BV (mgHA/cm^3^) for trabecular (MD-Tb) and cortical bone (MD-Cb).

#### 2.6.2. Radiographic Image Acquisition

**Figure 3:**
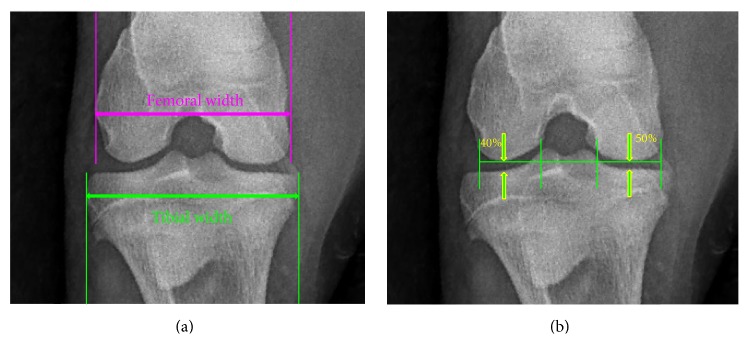
*Radiographic Image Acquisition*. The fixed-flexion weight-bearing stifle radiograph; showing the reference points for measuring joint width and medial and lateral tibiofemoral joint space widths (arrows). Width of the joint was recorded as the average of (a) the maximum width of the proximal tibia and (b) the maximum width of the distal femur. Maximum joint space width was measured as the maximum height of the joint space.

All sheep were handled in a routine manner using a halter and lead rope to achieve a balanced and fully weight-bearing, standing position for each radiographic study. Personnel wore appropriate radiation safety equipment (lead apron, lead gloves, and dosimetry badges) during each study. A myRad60 operating system (Universal Imaging, Bedford Hills, NY) and Poskom radiation generator (model: PXM-20BT, Diagnostic Imaging Systems, Inc., Rapid City, SD) were used for each study. Images were acquired with generator settings of 65 kV and 1.2 mAs. Image interpretation was performed using eFilm imaging software (Merge Healthcare, Chicago, IL). Three routine views and one special view were taken of both the left and right stifle at each imaging time point. The four total views consisted of (1) caudo 5° proximal-craniodistal oblique, (2) lateromedial, (3) caudo 60° lateral-craniomedial oblique, and (4) flexed-cranio-85° proximal-craniodistal oblique. From these images, measurements of joint width and joint space (mm), and the presence and severity of osteophytosis (0–4 scale), osteopenia (0–4 scale) and sclerosis (not evident during this study) were determined (Figure 3).

Width of the joint was recorded as the average of (a) the maximum width of the proximal tibia and (b) the maximum width of the distal femur. Maximum joint space width (JSW) was measured as the maximum height of the joint space. In the medial compartment, the width of the medial femoral condyle was determined and JSW was measured starting from the medial aspect of the condyle to a point equal to 50% of the total width of the condyle. In the lateral compartment, the width of the lateral condyle was determined and JSW was measured starting from the lateral aspect of the condyle to a point equal to 40% of the total width of the condyle (as shown in Figure 3). For both measures, the mean of the three measurements was recorded as joint width and JSW. All measurements were made by one author (HMM) to avoid interobserver variation and were assessed blind to treatment group.

### 2.7. Soft Tissue Analysis

#### 2.7.1. Urine and Serum Biomarkers

A modified 1,9-dimethylmethylene blue dye-binding assay (DMMB) was used on extracted urine samples to determine glycosaminoglycan (GAG) concentration as a marker of cartilage matrix degradation [14]. Extraction of GAG from urine samples included adding 1 M HCl to urine samples to pH 6.0 (approximately three drops/ml). Cetyltrimethylammonium bromide (50 g/L) was added to the samples (17 ul/ml) and the samples were stored at 4°C for 24 hr. Samples were then centrifuged at 5,000*g* for 15 min at 4°C; the pellet was washed with 95% ethanol and again stored at 4°C for 24 hr. The samples were then dried at 60°C and after the addition of 100 ul/ml 2 M NaCl, briefly vortexed, and centrifuged at 10,000*g* for 15 min at 4°C. The supernatant was then removed and 95% ethanol was added to the supernatant (500 ul/ml), which was briefly vortexed and stored at −20°C overnight. Samples were then centrifuged at 5,000*g* for 15 min at 4°C and the pellet was dried at 60°C, after which 50 ul/ml deionized water was added and the samples were lyophilized. We next added 50 ul/ml deionized water to the dried samples, mixed the samples well, then added 100 ul/ml acetone, and stored the samples at −20°C for 24 hr. Samples were then centrifuged at 5,000*g* for 15 min at 4°C, the pellet was then dried at 60°C, and the dried samples were used in the DMMB assay. Absorbance of the samples was determined at 525 nm, normalized to 690 nm, and plotted against a chondroitin sulfate-6 (CS) standard curve between 100 and 1000 ug/ml CS.

Concentrations of the aggrecan epitope CS846 were measured by a commercial enzyme-linked immunosorbent assay kit (ELISA, IBEX Diagnostics, Montreal, Quebec, Canada) as a marker of aggrecan synthesis (and degradation).

#### 2.7.2. Macroscopic Soft Tissue Grading

Macroscopic changes in cartilage were scored from grossly dissected stifle joints [15]. Assessment of cartilage included a score from 0–4 for each compartment (0 = normal, 1 = surface roughening, 2 = fibrillation and fissures, 3 = small erosions down to subchondral bone [<5 mm diameter], and 4 = larger erosions down to subchondral bone [>5 mm diameter]). Scores in all four compartments (medial tibial plateau, lateral tibial plateau, medial femoral condyle, and lateral femoral condyle) were totaled for a total cartilage score (0–16). Joints were well fixed and subjected to temperature changes during the micro-CT process. These conditions prevented accurate assessment of synovial pathology and thus no score is presented for synovial pathology.

### 2.8. Gait Analysis

**Figure 4:**
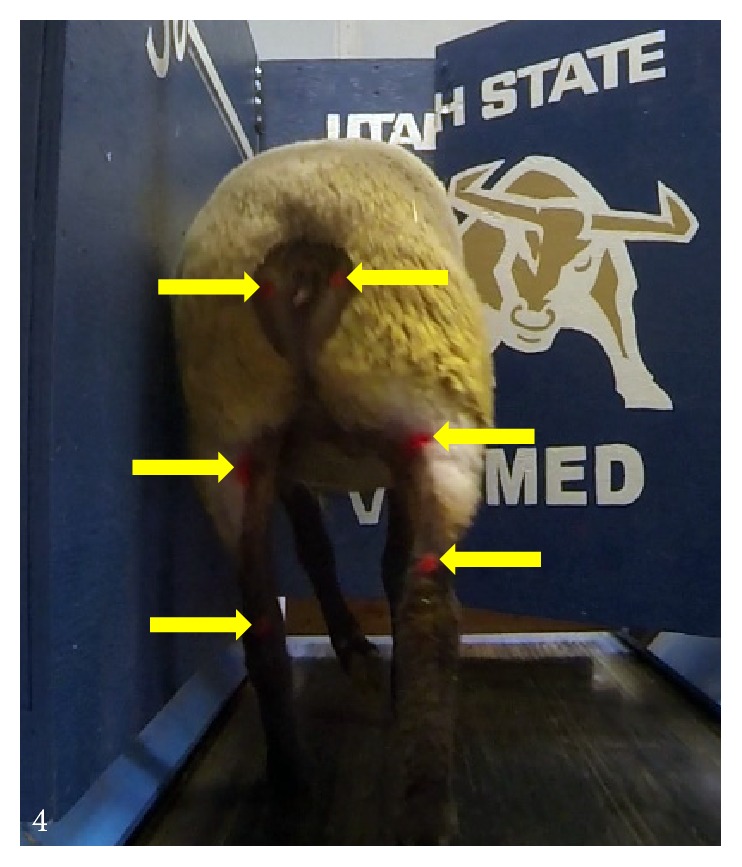
*Gait Analysis*. Sheep were trained to run on the treadmill two weeks prior to surgery. Painted markers can be seen at the Ischial tuberosity, tibial plateau, and calcaneal tuberosity. Destabilization surgery (CCLD) took place on Week 0. Treadmill exercise was initiated two weeks postoperatively on Week 2. Arrows indicate tracking points.

Frontal plane motion of the right leg was recorded for three minutes using a video camera (Casio Exilim EX-F1; 60 Hz with a shutter speed of .002 s) placed at a distance of 5.8 m from the object points and at a height of 0.8 m from the floor. The video camera was zoomed in so the image of the leg was as large as possible. Painted markers were applied to the following locations to aid in subsequent analysis of stifle angles: Ischial tuberosity, tibial plateau, and calcaneal tuberosity (Figure 4). Stifle angles for each condition were computed from the video images using a motion analysis program (Kinovea version 0.8.15). Specifically, relative stifle angles for each frame of motion for five gait cycles were computed with a digital goniometer that tracked the painted markers. The maximal stifle angle for each gait cycle was computed and the five-cycle average served as the outcome measure.

### 2.9. Statistical Analysis

Due to the small number of animals used in this pilot study, formal statistical analysis was not performed on most parameters measured, with the exception of Pearson correlations between osteophytosis grading methods and between exercise and MD-Tb values.

## 3. Results

### 3.1. Bone

#### 3.1.1. Micro-CT

Overall, the greatest changes in bone volume (BV and TV) were seen in the destabilized plus exercise group (Table 1 and Supplementary Tables S1–4 in Supplementary Material available online at https://doi.org/10.1155/2017/7481619).

The greatest changes in trabecular architecture and trabecular density were seen in the destabilized-only group (decreased). The exercise-only group displayed the greatest cortical bone density (increased). In the exercise-only group, changes were evenly divided between the lateral and medial sides and between the femoral and tibial compartments. In the destabilized animals that were not exercised, the greatest changes were seen in the medial versus the lateral compartments, with changes distributed fairly evenly between the femoral and tibial compartments. The greatest changes in the surgery plus exercise group were seen in the tibia, with changes divided fairly evenly between the lateral and medial plateaus.

#### 3.1.2. Digital Radiographs

**Figure 5:**
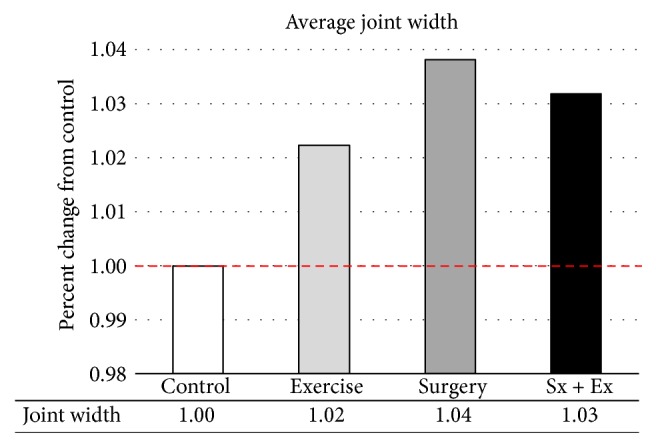
*Joint Width at the Point of Collection*. Joint width increased with all treatments. Increases were most severe in destabilized animals.

**Figure 6:**
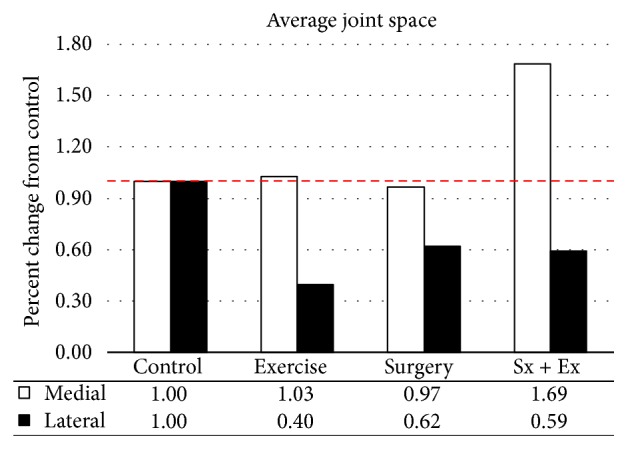
*Joint Space Changes from the Start of the Experiment to the Point of Collection*. Joint space decreased in the lateral compartment in all treatments but increased in the medial compartment of destabilized plus exercise group.

The width of the joint (average of the maximum width of the tibia and the maximum width of the femur at the articulating surface) increased with all treatments, compared with control sheep (Figure 5). Joint width increased twice as much in destabilized sheep (+4%) as in exercised sheep (+2%). Over time, joint space was decreased in the lateral, but not the medial side of the stifle and was increased in the medial compartment in the destabilized plus exercise animals (69%, Figure 6).

**Figure 7:**
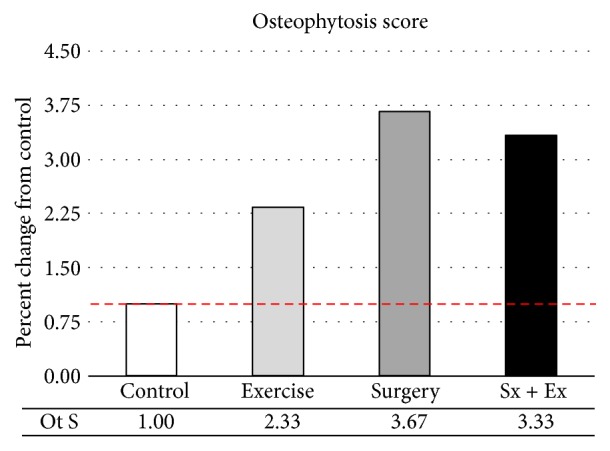
*Osteophytosis Score (0–4) at the Medial Tibial Plateau in the Left Joint*. Definitive osteophytosis was only detected at the medial tibial plateau in all groups and was increased by exercise and destabilization.

With digital radiography, osteophytosis was only detected at the medial tibial plateau and was increased with all treatments. Destabilized joints displaying the greatest increase in osteophytosis, compared with controls (Figure 7). Subchondral sclerosis, which normally occurs late in the disease process, was not radiographically evident during this study [16].

**Figure 8:**
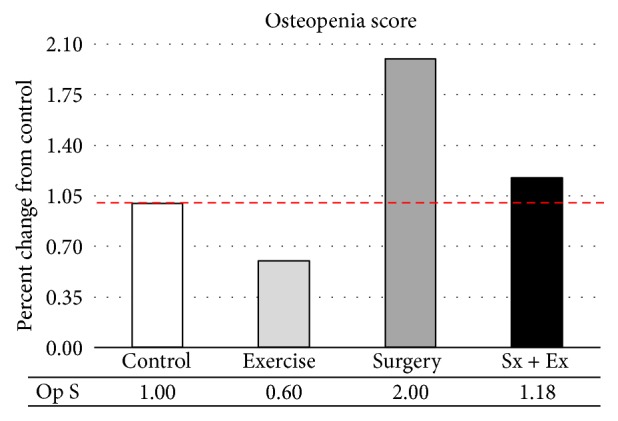
*Osteopenia Score (0–4) in All Four Quadrants Combined*. Osteopenia was decreased by exercise, increased by destabilization, and unchanged by destabilization plus exercise.

Reported osteopenia scores reflected the combination of the scores recorded for all four stifle compartments and were decreased with exercise and increased with destabilization (Figure 8). The exercise-induced decrease and destabilization-induced increase appeared to synergize and resulted in very little change in the destabilized plus exercise group.

### 3.2. Soft Tissue

#### 3.2.1. Urine GAG

**Figure 9:**
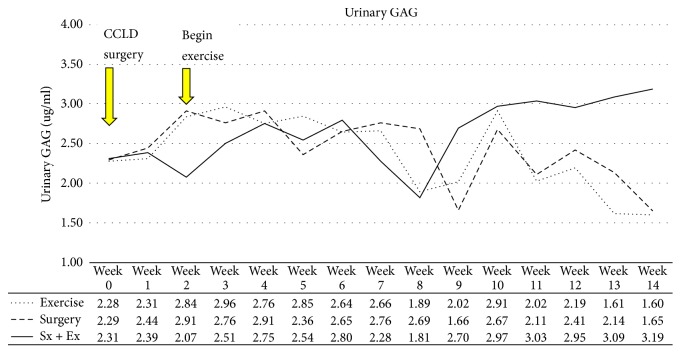
*Urinary GAG*. Exercise exacerbated the surgical effects on GAG breakdown.

Urinary GAG was mildly influenced similarly in all groups until Week 10 when the destabilized plus exercise group began increasing, while the destabilized-only and exercise-only groups were at baseline levels or below (Figure 9).

#### 3.2.2. Aggrecanase ELISA

**Figure 10:**
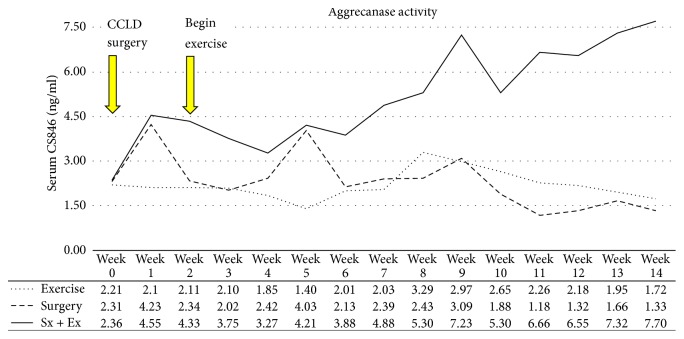
*Aggrecanase Activity*. Exercise significantly exacerbated the surgical effects on aggrecan breakdown.

Aggrecanase activity was initially increased by destabilization. This increase in aggrecanase activity returned to baseline levels in the destabilization-only group but continued to increase until the end of the study in the destabilized plus exercise animals. Exercise alone had no influence on aggrecanase activity (Figure 10).

#### 3.2.3. Macroscopic Scoring

**Figure 11:**
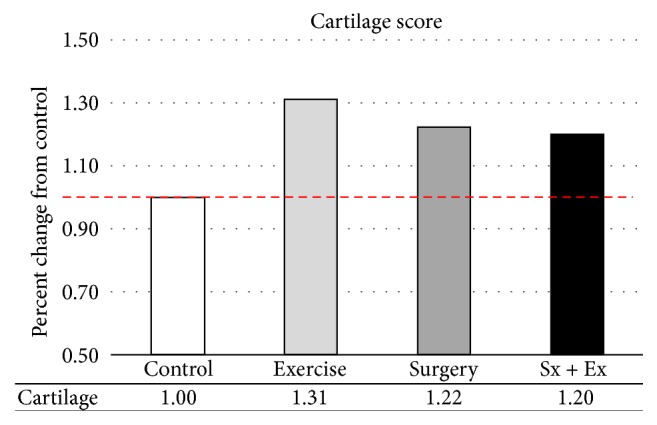
*Macroscopic Scoring*. Exercise and surgery both increased cartilage breakdown.

Cartilage damage was far more severe in the femoral condyles (average score = 3.58), compared with the tibial plateaus (average score = 1.75) (see Figure 11).

### 3.3. Gait Analysis

**Figure 12:**
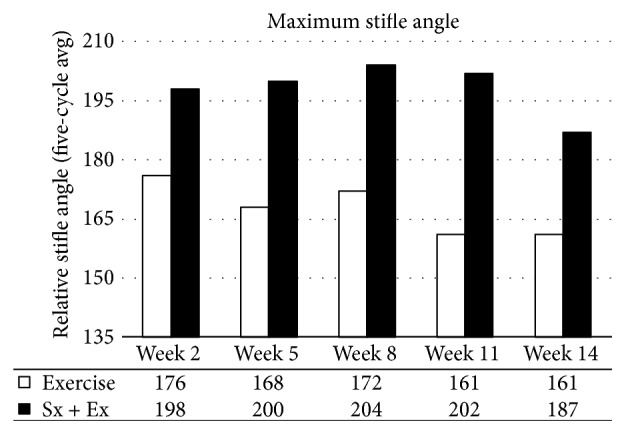
*Gait Analysis*. Surgery increased the maximum stifle angle and exercise decreased the maximum angle.

**Figure 13:**
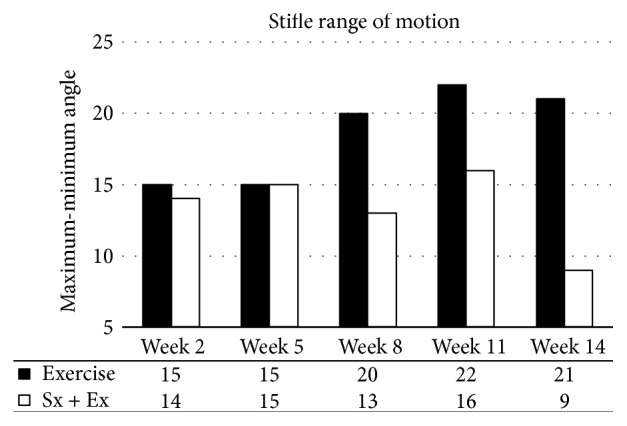
*Gait Analysis*. Exercise increased the range of motion and surgery decreased the range of motion.

Gait analysis was limited to one animal in the exercise-only group and one animal in the destabilized plus exercise group. Over time, exercise decreased the maximum stifle angle (Figure 12) and increased the range of motion (Figure 13). The destabilized plus exercise animal did not display major trends until the end of the experiment when maximum stifle angle and range of motion were both decreased.

## 4. Discussion

In the current experiments, surgical destabilization and forced exercise differed significantly in their influence on many of the parameters commonly associated with progression of OA. Not surprisingly, the combination of surgical destabilization and forced exercise synergistically influenced many of these OA parameters.

Ex vivo micro-CT was the most sensitive and quantitative measure of bone changes used in the current study and correlated well with results from digital radiographs. Large increases in BV and TV were induced by destabilization surgery. Forced exercise exacerbated these changes in destabilized animals but had little influence in stable animals. Because both BV and TV were increased, BV/TV was relatively unchanged in all groups. This pattern of increased BV and TV with no change in BV/TV reflects changes seen previously in the stifle joints of ovariectomized females but was opposite to results seen previously in a destabilized stifle model [17, 18]. In the current model, adding forced exercise to destabilized stifle joints produced bone volume changes more similar to stifle joints in ovariectomized versus destabilized animals.

The effect of increased cortical bone density in the exercise group and decreased trabecular bone density in the destabilized group reflect changes seen previously in both ovariectomized and destabilized OA models. These changes appeared to cancel each other out in the destabilized plus exercise group, which displayed cortical and trabecular bone density values very similar to controls. A destabilization-induced decrease in TbTh was also moderated by exercise in the destabilized plus exercise group, which displayed the same TbTh values seen in controls.

Unlike the ex vivo micro-CT analysis, with digital radiography, we were able to track changes over time and detected increases in the width of the stifle joint in all treatments, which were greatest in destabilized joints. Not unexpectedly, changes in joint width correlated very closely with changes in osteophytosis (Pearson correlation = 0.991). Previously, osteophytosis grading from micro-CT 3D reconstructed images in a destabilized stifle model produced scores corresponding to a 3.56% change from control joints. In the current experiments, osteophytosis grading from digital radiographs of our destabilized stifle model produced scores corresponding to a 3.67% change from control joints. This observation suggests that data from micro-CT 3D reconstructed images and from digital radiographs may be interpreted with a similar level of confidence.

Joint space narrowing is a common measure of OA progression and is often detected with radiography in human medicine. In the current report, joint space narrowing was detected in the lateral, but not the medial compartment of the stifle joint. In fact, joint space was increased in the medial compartment in the destabilized plus exercise group. This appears to contradict the observed decrease in range of motion and decreased maximum stifle angle detected in the gait analysis. However, the gait analysis only included one animal per group and should be interpreted with caution. Exercise had a positive influence on gait analysis producing a decreased maximum stifle angle and an increased range of motion, suggesting increased control of the joint over time. Exercise may have had a positive influence on the destabilized plus exercise animal, which seemed to display increasing maximum stifle angle until the end of the experiment, at which point the maximum angle was decreased, although the range of motion in the stifle was decreased as well in this animal.

Osteopenia, normally present in ovariectomized and destabilized animal models, was decreased by exercise, increased by destabilization, and unchanged in the destabilized plus exercise model. These results again correlate well with the micro-CT, MD-Tb values and we again see the effects of exercise positively influencing bone density in the destabilized joint (Pearson correlation = 0.938).

In the current experiments, exercised often countered the negative effects of destabilization surgery on bone changes associated with OA progression. However, in the cartilage, exercise appeared to exacerbate the negative effects of destabilization surgery.

Degenerative breakdown of the components of the cartilage matrix, including GAGs, results in the generation of fragments of these cartilage macromolecules, which can eventually be detected in the urine. Cartilage contains the highest concentration of GAGs of any mammalian tissue and most urinary GAGs are of systemic origin [19]. Athletic horses excrete less GAGs in the urine than sedentary horses [20]. Urinary GAG varied little until the end of our experiments, when exercise pushed urinary GAG levels higher in destabilized plus exercise animals, but the exercise-only and destabilized-only animals displayed urinary GAG levels at baseline or below. Our methods concentrated the GAG content of the urine in relation to other urine components, but the use of DMMB did not allow discrimination of any contaminating substances present. This assay was intended for use as a screening assay to be followed up with subsequent detection methods when indicated.

Aggrecan is a large, aggregating proteoglycan and possesses a core protein with covalently attached sulfated GAG chains. Increased aggrecanase activity is often seen as a measure of increased cartilage degeneration. Aggrecanase activity is normally very low in adults, but detection of aggrecan fragments/neoepitopes is increased in the serum of horses with joint disease [21]. In the current experiments, exercise alone had no influence on aggrecanase activity. However, when applied to a destabilized joint, exercise prevented the return to baseline levels of the early destabilization-induced spike of aggrecanase activity (seen in the destabilized-only joints) but instead induced further increases in aggrecanase activity to the end of the experiments. As with urinary GAG, exercise exacerbated the negative effects of destabilization on aggrecan breakdown.

Macroscopic scoring of cartilage added further support for the negative influence of forced exercise on cartilage degradation. However, exercise did not appear to be additive with the effects of surgery on soft tissue breakdown as scored macroscopically.

In summary, the current pilot experiments demonstrated that our large animal OA model was able to produce clinically detectable signs of OA in a relatively short time frame. Forcing the sheep to run on the treadmill at an oblique angle likely contributed to the relatively rapid onset of clinical signs by increasing stress on the stifle joint. However, we did not include a control group that ran on the treadmill in a conventional, inline running protocol. Changes in bone were highly correlated between micro-CT and radiographic analysis and changes in cartilage correlated well between urinary GAG and serum aggrecanase analyses and macroscopic scoring. However, the effects of exercise in destabilized stifle joints differed significantly between soft tissue and bone. Exercise often improved the negative effects of destabilization in bone, but at the same time exacerbated the negative effects of destabilization in cartilage. These results represent an improved large animal model of osteoarthritis with rapid onset of disease and superior detection of bone and soft tissue changes.

## Supplementary Material

Supplemental Tables include data collected from each compartment of the stifle joint, including Lateral Femoral (S1), Medial Femoral (S2), Lateral Tibial (S3) and Medial Tibial (S4) compartments.

## Figures and Tables

**Table 1:** Effect of exercise, surgery, and surgery + exercise, *average of all compartments *(mean ± SD).

Treatment group	Control	Exercise	(%)	Surgery	(%)	Surgery + exercise	(%)
BV (mm^3^)	1364 ± 164	1361 ± 174	(−0.2)	1474 ± 319	(8.1)	1700 ± 11.60	(24.6)
TV (mm^3^)	2141 ± 340	2128 ± 296	(−0.6)	2327 ± 555	(8.7)	2626 ± 19.24	(22.6)
BV/TV (%)	0.160 ± 0.006	0.160 ± 0.007	(0.5)	0.159 ± 0.006	(−0.5)	0.162 ± 0.002	(1.6)
BS/BV (mm^−1^)	6.56 ± 0.247	6.67 ± 0.208	(1.7)	6.74 ± 0.046^a^	(2.8)	6.56 ± 0.008^b^	(0.1)
TbTh (mm)	0.307 ± 0.012	0.302 ± 0.010	(−1.6)	0.298 ± 0.001^a^	(−2.7)	0.305 ± 0.00^b^	(−0.5)
TbN (mm^−1^)	1.98 ± 0.028	2.01 ± 0.053	(1.6)	1.99 ± 0.051	(0.6)	1.98 ± 0.019	(0.2)
TbSp (mm)	0.200 ± 0.011	0.197 ± 0.015	(−1.7)	0.204 ± 0.015	(2.1)	0.198 ± 0.004	(0.8)
TbPf (mm^−1^)	−4.51 ± 0.581	−4.66 ± 0.820	(3.2)	−4.48 ± 0.318	(−0.7)	−4.54 ± 0.135	(0.6)
MD-Tb (mgHA/cm^3^)	202 ± 11.7	208 ± 4.90	(2.7)	185 ± 14.3	(−8.8)	191 ± 9.04	(5.4)
MD-Cb (mgHA/cm^3^)	297 ± 32.2	338 ± 10.8^a^	(13.7)	280 ± 24.6^b^	(−5.7)	289 ± 4.94^b^	(2.6)

^a, b^Different alphabetic superscripts signify values significantly different from one another (*P* < 0.05).

(%): percent change from Control joint.
