# Supplementary material for: The Influence of Oblique Angle Forced Exercise in Surgically Destabilized Stifle Joints Is Synergistic with Bone, but Antagonistic with Cartilage in an Ovine Model of Osteoarthritis
Source: Arthritis. 2017 Feb 27;2017:7481619. doi: 10.1155/2017/7481619 (PMC5350401; doi:10.1155/2017/7481619)
Supplement: Supplementary file 1 [file 7481619.f1.docx]

Table S1. Effect of exercise, surgery and surgery + exercise - ***Lateral femoral compartment*** (mean values +/- SD).

Treatment group	Control	Exercise	(%)	Surgery	(%)	Surgery + Exercise	(%)
BV (mm^3)	997 +/- 50.3[a]	1109 +/- 122	(11.2)	1072 +/- 142	(7.5)	1158 +/- 71.8[b]	(16.1)
TV (mm^3)	1490 +/- 99.0	1654 +/- 154	(11.0)	1651 +/- 323	(10.8)	1667 +/- 78.4	(11.9)
BV/TV (%)	0.670 +/- 0.020	0.670 +/- 0.023	(0.2)	0.655 +/- 0.041	(-3.0)	0.694 +/- 0.017	(3.7)
BS/BV (mm^{-1})	6.88 +/- 0.297	6.78 +/- 0.490	(-1.5)	6.83 +/- 0.396	(0.7)	6.40 +/- 0.159	(-7.0)
TbTh (mm)	0.293 +/- 0.015	0.297 +/- 0.025	(1.1)	0.293 +/- 0.015	(0.0)	0.313 +/- 0.006	(6.8)
TbN (mm^{-1})	2.12 +/- 0.032[a]	2.10 +/- 0.110	(-0.9)	2.08 +/- 0.035	(-2.2)	2.04 +/- 0.015[b]	(-3.8)
TbSp (mm)	0.180 +/- 0.010	0.183 +/- 0.015	(1.9)	0.187 +/- 0.012	(3.7)	0.173 +/- 0.006	(-3.7)
TbPf (mm^{-1})	-4.73 +/- 0.477	-4.72 +/- 0.510	(-0.4)	-4.79 +/- 0.353	(1.3)	-4.85 +/- 0.153	(2.4)
MD-Tb ($mgHA/cm^3$)	204 +/- 34.3	224 +/- 16.3	(9.8)	193 +/- 6.61	(-5.3)	207 +/- 23.0	(1.4)
MD-Cb ($mgHA/cm^3$)	311 +/- 52.5	364 +/- 6.31[a]	(16.8)	299 +/- 23.7[b]	(-4.0)	305 +/- 13.1[b]	(-1.9)

a,bDifferent alphabetic superscripts signify values significantly different from one another (P<0.05).
(%)-percent change from Control joint

Table S2. Effect of exercise, surgery and surgery + exercise -**Medial femoral *compartment*** (mean values +/- SD).

Treatment group	Control	Exercise	(%)	Surgery	(%)	Surgery + Exercise	(%)
BV (mm^3)	1154 +/- 120	1127 +/- 110	(-2.3)	1209 +/- 221	(4.8)	1289 +/- 105	(11.7)
TV (mm^3)	1613 +/- 197	1606 +/- 208	(-0.4)	1725 +/- 347	(6.9)	1826 +/- 142	(13.2)
BV/TV (%)	0.716 +/- 0.014	0.704 +/- 0.029	(-1.8)	0.703 +/- 0.015	(-2.0)	0.706 +/- 0.011	(-1.3)
BS/BV (mm^{-1})	5.95 +/- 0.124	6.19 +/- 0.061	(4.0)	6.16 +/- 0.410	(3.5)	6.15 +/- 0.150	(3.2)
TbTh (mm)	0.333 +/- 0.006	0.323 +/- 0.006	(-3.0)	0.327 +/- 0.025	(-2.0)	0.323 +/- 0.006	(-3.0)
TbN (mm^{-1})	2.01 +/- 0.006	2.05 +/- 0.062	(2.2)	2.01 +/- 0.100	(0.0)	2.04 +/- 0.040	(1.5)
TbSp (mm)	0.163 +/- 0.012	0.163 +/- 0.015	(0.0)	0.173 +/- 0.006	(6.1)	0.167 +/- 0.006	(2.0)
TbPf (mm^{-1})	-5.50 +/- 0.452	-5.58 +/- 0.740	(1.5)	-5.45 +/- 0.492	(-0.8)	-5.51 +/- 0.321	(0.2)
MD-Tb ($mgHA/cm^3$)	241 +/- 11.9	243 +/- 18.8	(0.7)	215 +/- 12.1	(-11.1)	223 +/- 5.13	(-7.6)
MD-Cb ($mgHA/cm^3$)	324 +/- 38.5	363 +/- 24.5[a]	(12.0)	296 +/- 17.6[b]	(-8.8)	317 +/- 4.06	(-2.1)

a,bDifferent alphabetic superscripts signify values significantly different from one another (P<0.05).
(%)-percent chnage from Control joint

Table S3. Effect of exercise, surgery and surgery + exercise - ***Lateral tibial compartment*** (mean values +/- SD).

Treatment group	Control	Exercise	(%)	Surgery	(%)	Surgery + Exercise	(%)
BV (mm^3)	1646 +/- 600	1634 +/- 257[a]	(-0.7)	1885 +/- 636	(14.5)	2409 +/- 174[b]	(46.4)
TV (mm^3)	2830 +/- 1192	2654 +/- 420[a]	(-6.2)	3126 +/- 1063	(10.5)	3880 +/- 274[b]	(37.1)
BV/TV (%)	0.591 +/- 0.036	0.616 +/- 0.028	(5.9)	0.603 +/- 0.021	(3.7)	0.621 +/- 0.007	(6.8)
BS/BV (mm^{-1})	7.11 +/- 0.729	6.91 +/- 0.288	(-2.8)	7.12 +/- 0.156	(0.1)	6.86 +/- 0.067	(-3.6)
TbTh (mm)	0.283 +/- 0.032	0.290 +/- 0.010	(2.4)	0.280 +/- 0.010	(-1.2)	0.290 +/- 0.000	(2.4)
TbN (mm^{-1})	1.94 +/- 0.104	1.97 +/- 0.040	(1.4)	1.95 +/- 0.075	(0.3)	1.92 +/- 0.040	(1.0)
TbSp (mm)	0.233 +/- 0.023	0.217 +/- 0.015	(-7.1)	0.233 +/- 0.021	(0.0)	0.230 +/- 0.010	(-1.4)
TbPf (mm^{-1})	-3.67 +/- 0.755	-4.20 +/- 1.03	(14.4)	-3.70 +/- 0.658	(0.8)	-3.95 +/- 0.366	(7.6)
MD-Tb ($mgHA/cm^3$)	165 +/- 15.4	164 +/- 10.0	(-0.6)	151.7 +/- 33.5	(-8.3)	151.2 +/- 5.65	(-8.6)
MD-Cb ($mgHA/cm^3$)	256 +/- 35.5	286 +/- 18.1	(11.5)	243.6 +/- 34.4	(-4.9)	252.7 +/- 16.7	(-1.3)

a,bDifferent alphabetic superscripts signify values significantly different from one another (P<0.05).
(%)-percent chnage from Control joint

Table S4. Effect of exercise, surgery and surgery + exercise - ***Medial tibial compartment*** (mean values +/- SD).

Treatment group	Control			Exercise			(%)	Surgery			(%)	Surgery + Exercise			(%)
BV (mm^3)	1658	+/-	120	1575	+/-	216	(-5.0)	1731	+/-	325	(4.4)	1943	+/-	257	(17.2)
TV (mm^3)	2632	+/-	223	2597	+/-	421	(-1.3)	2805	+/-	612	(6.6)	3132	+/-	449	(19.0)
BV/TV (%)	0.631	+/-	0.023	0.609	+/-	0.030	(-3.7)	0.621	+/-	0.05	(-2.1)	0.621	+/-	0.008	(-1.5)
BS/BV (mm^{-1})	6.29	+/-	0.317	6.79	+/-	0.499	(8.1)	6.85	+/-	0.55	(8.9)	6.85	+/-	0.174	(8.9)
TbTh (mm)	0.317	+/-	0.015	0.297	+/-	0.023	(-6.3)	0.293	+/-	0.03	(-7.4)	0.293	+/-	0.006	(-7.4)
TbN (mm^{-1})	1.85	+/-	0.020	1.93	+/-	0.052	(4.3)	1.94	+/-	0.04	(4.7)	1.93	+/-	0.090	(4.5)
TbSp (mm)	0.223	+/-	0.012	0.223	+/-	0.021	(0.0)	0.223	+/-	0.03	(0.0)	0.223	+/-	0.015	(0.0)
TbPf (mm^{-1})	-4.15	+/-	0.724	-4.13	+/-	1.10	(-0.6)	-3.99	+/-	0.84	(-3.9)	-3.85	+/-	0.344	(-7.2)
MD-Tb ($mgHA/cm^3$)	199	+/-	6.98	201	+/-	21.9	(0.7)	179	+/-	23.06	(-10.1)	185	+/-	7.51	(-7.2)
MD-Cb ($mgHA/cm^3$)	297	+/-	17.3	339	+/-	45.6	(14.1)	283	+/-	37.56	(-4.6)	281	+/-	17.7	(-5.1)

a,bDifferent alphabetic superscripts signify values significantly different from one another (P<0.05).

(%)-percent chnage from Control joint
